# Peters anomaly with post axial polydactyly, bilateral camptodactyly and club foot in a Kenyan neonate: a case report

**DOI:** 10.1186/1752-1947-6-16

**Published:** 2012-01-17

**Authors:** Aruyaru Stanley Mwenda

**Affiliations:** 1 Department of Paediatrics, Consolata Hospital Mathari, P.O Box 25 00100 Nyeri, Kenya

## Abstract

**Introduction:**

A case of bilateral Peters anomaly with bilateral post axial polydactyly, bilateral camptodactyly, and club foot was examined in a neonatal Kenyan baby girl of African descent who had been delivered in the hospital and admitted to the newborn unit. She died aged five days. There are no cases of Peters anomaly recorded in Africa according to a literature search. In addition, available data point to the majority of the principal associations in Peters anomaly to be genitourinary anomalies, making this case a rare one in its isolated collection of musculoskeletal associations.

**Case presentation:**

A Kenyan baby girl of African descent who was born through a caesarean section presented in the new born unit of our hospital with bilateral corneal opacities, bilateral polydactyly, camptodactyly and club foot.

**Conclusion:**

This is a rare case of Peters anomaly and its association with multiple musculoskeletal abnormalities makes it special.

## Introduction

Peters anomaly is one of the main causes of congenital corneal opacities. It is a rare form of anterior chamber development, either sporadic or inherited [1,2], that presents as corneal opacity from birth with the opaque cornea obstructing the pupil and thus causing visual loss. In addition, there is anterior chamber dysgenesis with connection between the cornea and the iris and/or the lens in some cases.

## Case report

A Kenyan baby girl of African descent was delivered at term through an emergency caesarean section due to fetal distress (meconium stained liquor grade II with mild uterine contractions at cervical dilatation of 7 cm.). The mother had only received iron supplements during pregnancy and did not have an obstetric ultrasound done. The elder sibling was delivered vaginally and did not have any anomalies. There was no family history of any congenital anomalies.

The neonate was noted to have clouded cornea. Also noted in the cesarean section room was the presence of extra digits and club foot.

As a normal procedure, the baby was received at our newborn unit for stabilization and observation while the mother recuperated from anesthesia.

**Figure 1:**
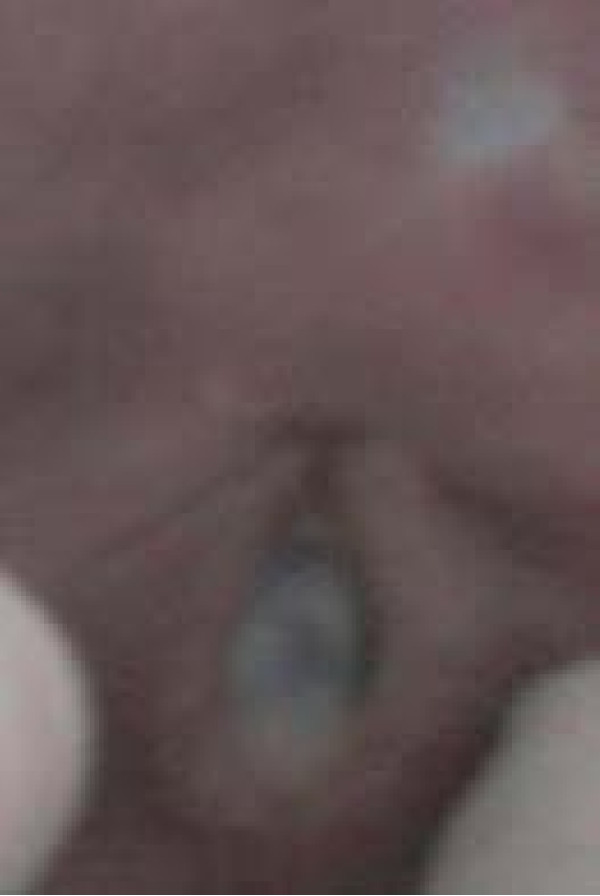
**Corneal opacification, right eye**.

**Figure 2:**
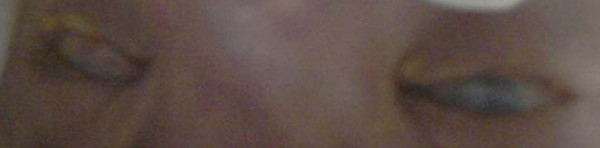
**Bilateral corneal opacification**.

On examination the baby was noted to have bilateral corneal opacities which were completely covering the iris and pupils (see Figures 1 and 2). The opacities were totally homogeneous with no blood vessels visible. The anterior chambers appeared normal with no strands between the cornea and the iris or lens.

**Figure 3:**
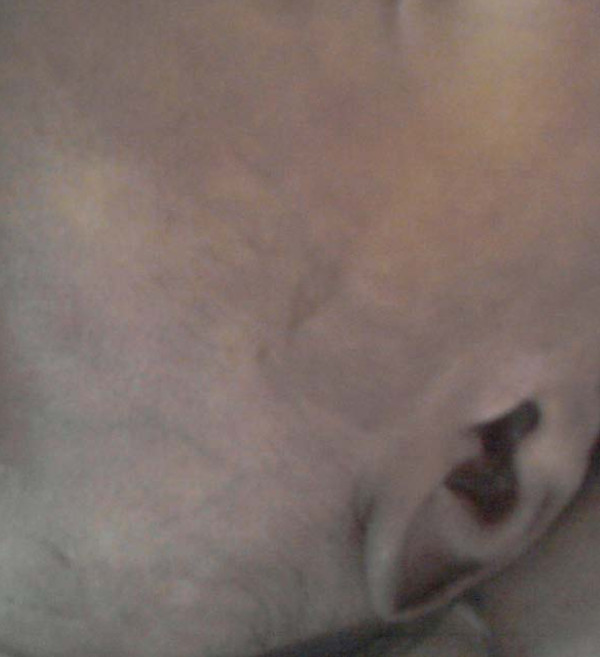
**Low set ears**.

**Figure 4:**
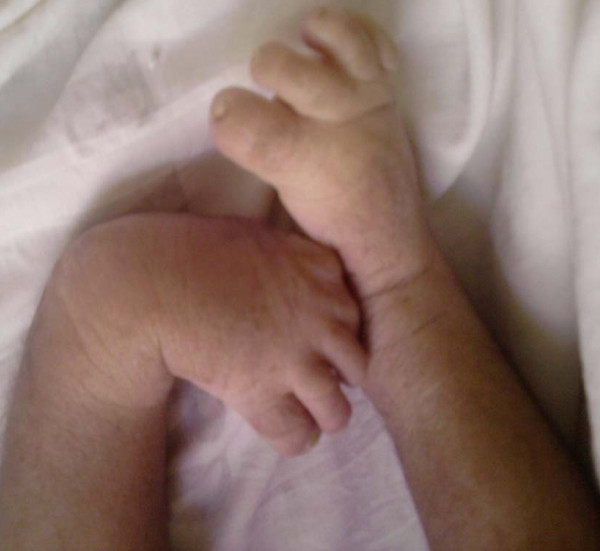
**Congenital Talipes Equinovarus of the left foot**. Note the excessive lanugo on the skin.

**Figure 5:**
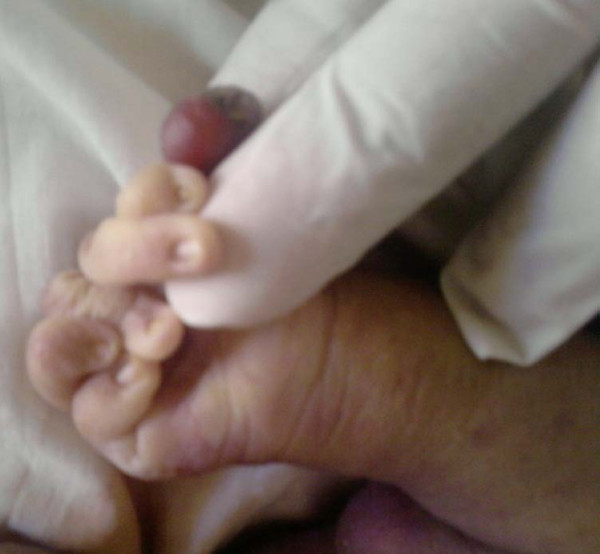
**Extra digit, Camptodactyly left upper limb**.

**Figure 6:**
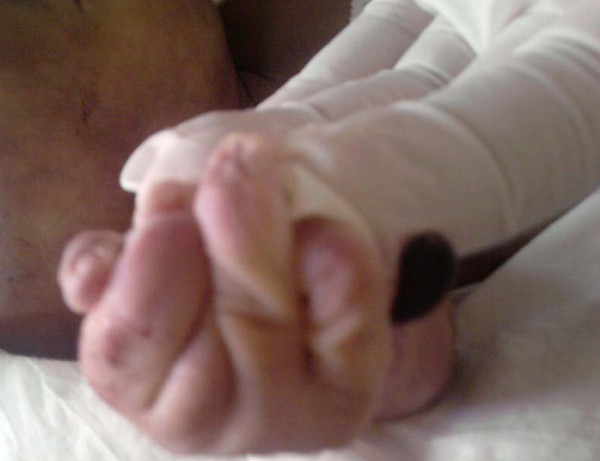
**Extra digit, Camptodactyly right upper limb**.

Other findings included low set ears with poorly developed cartilage on the pinna, flexion of the distal inter phalangeal joint of the forefinger and the proximal interphalangeal joints of the middle finger bilaterally. There was complete congenital talipes equinovarus of the left foot and varus deformity of the right foot (see Figures 3, 4, 5 and 6). There were no genital, vertebral, cardiac or tracheoesophageal anomalies on physical examination. The absence of congenital atresia of the esophagus was confirmed by the passage of a size 5 nasogastric tube into the stomach without resistance and aspiration of gastric contents. The neonate was also passing meconium well and did not develop any chocking or cyanotic spells on feeding. No radiological investigations were done due to financial prioritization.

In the course of management the baby developed ophthalmia neonatorum and neonatal sepsis and later died during day five of life. Although the parent had agreed to a post mortem examination, the relatives later decided otherwise and buried the body without having a post mortem examination and thus the cause of death could not be established. 

## Discussion

Peters anomaly is a developmental disorder of the cornea which can occur either sporadically (in most of the cases) or be inherited in a few cases. Inherited cases are either autosomal dominant or recessive [1]. It is among the causes of congenital clouding and/or opacification of the cornea. Other causes of congenital corneal opacification include sclerocornea, tears in the Descemet membrane secondary to birth trauma, ulcers, congenital glaucoma, congenital hereditary endothelial damage and various metabolic derangements [2]. It is important to differentiate Peters anomaly from other causes of corneal opacification. In Peters anomaly, the corneal opacity is either central or paracentral and it usually does not exhibit vascularization [3-5].

Two forms of Peters anomaly are recognized. Type I which occurs in the majority of the cases and type II which occurs less often [2]. In the type I anomaly, the lens may or may not be cataractous and does not adhere to the cornea. Type II is usually associated with a cataractous lens. The lens in this type also adheres to the cornea [2-4]. Normally mutations in the PAX6 gene are associated with defects in the development of ocular tissues [5]. Thus, some cases of Type II Peters anomaly, which often tends to occur bilaterally, may be associated with PAX6 gene mutations [4-6]. This type is also associated with more systemic and ocular malformations [4].

The etiology of Peters anomaly is not known and environmental as well as genetic factors are thought to play a role in its genesis [5]. The critical step in the development of Peters anomaly occurs in the first trimester during the formation of the anterior chamber [5].

The few published studies do not show any gender predilection for Peters anomaly. A study by Bhandari *et al.*, for instance, showed equal distribution in both sexes [6]. More cases tend to occur bilaterally [6] and these are more prone to have systemic associations [6]. However, both types of Peters anomaly may be either unilateral or bilateral. Among the systemic associations occurring with Peters anomaly are congenital heart disease, neurologic defects, genitourinary abnormalities, external ear abnormalities and cleft lip and palate [4]. Various chromosomal abnormalities have also been associated with Peters anomaly among them trisomy 13-15, ring chromosome 21, Norrie disease, partial deletion of chromosome 11 q, mosaic trisomy 9 and the 49XXXXY syndrome [4] ( see Table 1).

**Table 1:** Published associations in Peters anomaly

AUTHORS	YEAR	TITLE	CONCULSION
Shanske AL *et al.*	2002	Possible new syndrome of microcephaly with cortical migration defects, Peters anomaly and multiple intestinal atresias: a multiple vascular disruption syndrome [10]	New multiple associations-nervous and gastrointestinal
Banning CS *et al.*	2005	Corneal perforation with secondary congenital aphakia in Peters anomaly [11].	Ocular association
Neilan E *et al.*	2006	Peters anomaly in association with multiple midline anomalies and a familial chromosome 4 inversion [12]	New multiple associations- orofacial, cardiac, nervous
Navneet T *et al.*	2009	Peters plus syndrome and absence of kidney: a case report [13]	New association- unilateral renal agenesis
Aliferis K *et al.*	2010	A novel nonsense B3GALTL mutation confirms Peters plus syndrome in a patient with multiple malformations and Peters anomaly [14]	Confirmed specific mutation in peters plus syndrome
Shimizu R *et al.*	2010	Severe Peters plus syndrome-like phenotype with anterior eye staphyloma and hypoplastic left heart syndrome: proposal of a new syndrome [15]	New multiple associations-ocular and cardiovascular
Lim Z, Quah BL	2010	Unilateral retinoblastoma in an eye with Peters anomaly [16]	Ocular association and a specific gene mutation
Arikawa A *et al.*	2010	Case of novel PITX2 gene mutation associated with Peters' anomaly and persistent hyperplastic primary vitreous [17]	Ocular association and a specific gene mutation
Sawada M *et al.*	2011	A case of aniridia with unilateral Peters anomaly [18]	New association- aniridia Genetic link- PAX 6 mutation

Recorded attempts to treat Peters anomaly involve keratoplasty. The success rate, which relies on strict amblyopia therapy and requires prolonged follow-up, varies but is better with isolated unilateral cases [6,7]. 

Our case has its unique characteristics. With a normal anterior chamber and lens, and the absence of adherence between the cornea and the lens, it seems to be type I Peters anomaly. However, its bilaterality and multiple associations argue for a unique association not recorded elsewhere in literature. The lost opportunity to do a post mortem study also denied the author the chance to find (or rule out) other systemic associations. Furthermore, as noted above, the systemic associations with Peters anomaly tend to be genitourinary, neurologic, cardiac and orofacial [4,6]. The prominence of musculoskeletal associations in this case makes it a special one and points to a possible new syndrome. Low set ears are a feature associated with the trisomies [8,9] and it is regrettable that it was not possible to rule in or out a genetic link to the defect. The author fully acknowledges that this could be pure coincidence, though!

## Conclusions

A case report is presented of a neonate with multiple congenital anomalies that do not fit into one established diagnostic entity although there were shortcomings in the work up of our patient and there still may remain gray areas in the final diagnosis. Such rare cases need to be reported to enhance further study and characterization. This is a case of bilateral Peters anomaly with unusual multiple musculoskeletal associations.

## Consent

Written informed consent was obtained from the patient's next-of-kin for publication of this case report and any accompanying images. A copy of the written consent is available for review by the Editor-in-Chief of this journal.

## Competing interests

The author declares that he has no competing interests.
